# Supplementary material for: Mediation analysis in longitudinal intervention studies with an ordinal treatment-dependent confounder
Source: Stat Methods Med Res. 2026 Mar 18;35(4):773–94. doi: 10.1177/09622802261418211 (PMC13161496; doi:10.1177/09622802261418211)
Supplement: sj-pdf-1-smm-10.1177_09622802261418211 - Supplemental material for Mediation analysis in longitudinal intervention studies with an ordinal treatment-dependent confounder [file sj-pdf-1-smm-10.1177_09622802261418211.pdf]

Supplementary material to Mediation analysis in longitudinal intervention studies with an ordinal treatment-dependent confounder

Journal Title
XX(X):1–6
©The Author(s) 0000
Reprints and permission:
sagepub.co.uk/journalsPermissions.nav
DOI: 10.1177/ToBeAssigned
www.sagepub.com/
SAGE

S1 Alternative models for treatment-dependent confounder

To assess whether a more flexible parameterisation of the model for the treatment-dependent confounder should be preferred, we also considered models with interaction between the treatment and the baseline covariates. The models were compared using Pareto smoothed importance sampling leave-one-out cross validation[2]. The differences in the expected log pointwise predictive masses and their standard errors are shown in Table 1. As there was no indication in favour of any of the more flexible parameterisations, the model with no interaction effects (i.e. model (7) of the main text) was chosen.

Supplementary Table 1. Results of the model comparison. The column 'ELPPM difference' refers to the difference in the expected log pointwise predictive mass as compared to the best performing model (i.e., one with interaction between treatment and sex) and 'SD' to the standard deviation of the difference. The model 'none' refers to the simplest model containing no interactions and is here preferred since none of the more complex models could be shown to outperform it.

Interactions with treatment	ELPPM difference	SD
sex	0	0
none	-0.1	2.1
age	-1.8	3.6
score at baseline	-3.5	2.7
smoke	-4.0	2.4
all	-7.9	3.7

The proportions of posterior samples failing the monotonicity assumption (see Eq. (3) in the main text) in each of the compared models are shown in Figure 1. As there were quite many strata with a large proportion of posterior samples failing the monotonicity condition under the model with all interactions, we chose to conduct a sensitivity analysis estimating lower and upper bounds for the mediational effects under this model and relaxing the monotonicity assumption[3]. In addition, as current smokers appeared to prominently feature in the strata challenging the monotonicity assumption, we also performed a sensitivity analysis dropping current smokers.

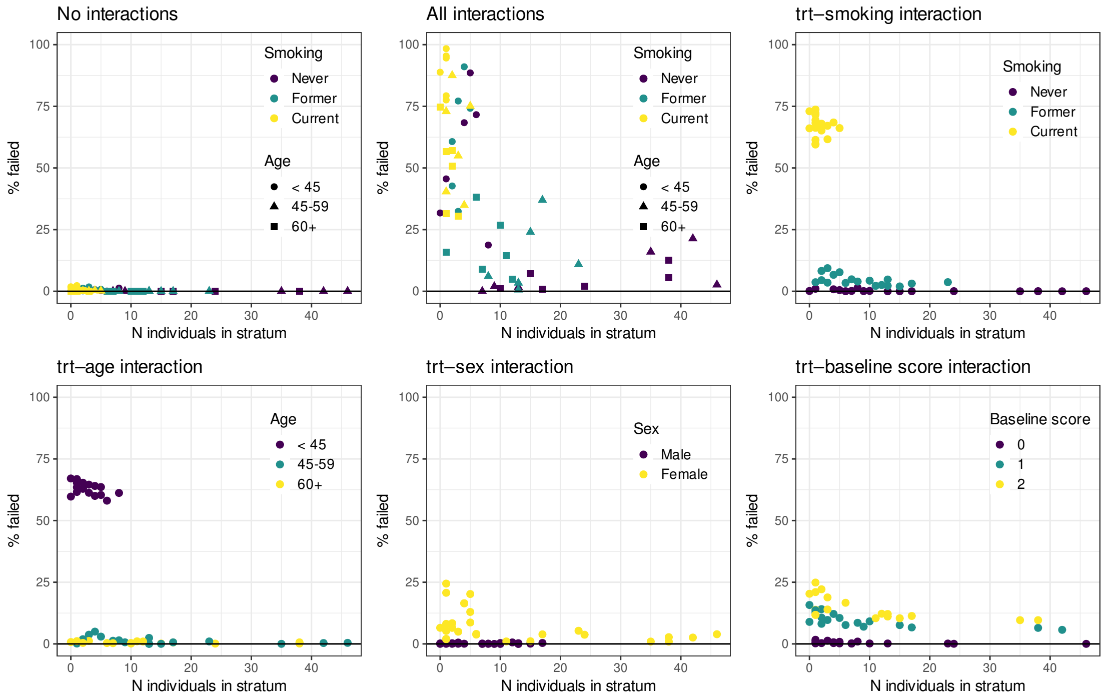

Supplementary Figure 1. Proportions of posterior samples failing the monotonicity condition under the considered models for the treatment-dependent confounder.

S2 Joint posterior distributions of the causal effects

The joint posterior distributions of the total, direct and indirect effects are shown in Figure 2. Under the three-year legacy parameterisation, the direct and indirect effects have a strong negative correlation while under the current change parameterisation no appreciable correlation is observed. The total effect is strongly correlated with the direct effect, representing the fact that the variance of the direct effect is much larger than the variance of the indirect effect.

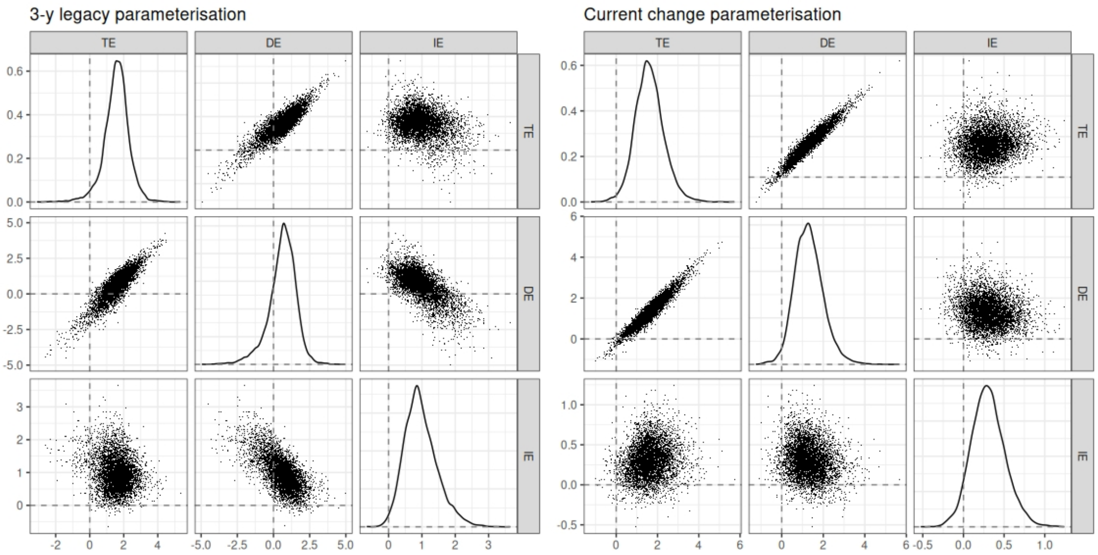

Supplementary Figure 2. Pairwise joint posterior distributions of the total (TE), direct (DE) and indirect (IE) effects under the three-year legacy and the current change parameterisations.

S3 Estimation procedure

Figure 3 illustrates the steps used to estimate the total, direct and indirect effects. The parameters $\boldsymbol{\theta}$ in the models of the repeated measurement (BMI $M(\cdot)$) and survival (diabetes-free time T) were estimated jointly (Step 1), as the two models shared some of the parameters. The parameters $\boldsymbol{\phi}$ determining the probabilities for the treatment-dependent confounder were estimated separately from the joint model (Step 2). Figure 3 corresponds to Figure 1 in the main text, indicating (via dashed boxes) that the model for the nodes T and $M(\cdot)$ was estimated separately from the model for L. Fitted under the Bayesian paradigm using the Stan software, we produced 8000 samples from four parallel chains of the two posterior distributions of the model parameters.

The parametric identification formulae (see section 3.4 in the main text) were evaluated by substituting the model parameters. Applying this procedure to each of the 8,000 posterior samples (Step 3) yielded 8,000 samples from the joint posterior distributions of the causal effects. The point estimates were computed as the means of the posterior samples and the credible intervals as the 2.5% and 97.5% quantiles.

S4 Simulations

To test the implementation of our estimation method and to assess how its performance depends on sample size, we simulated datasets with 100, 300, 500 and 1000 hypothetical individuals and compared the point estimates (posterior means) of the total, direct and indirect effects with the sample-based "true" effects. For each scenario, we generated 100 independent complete-data replications. Data were

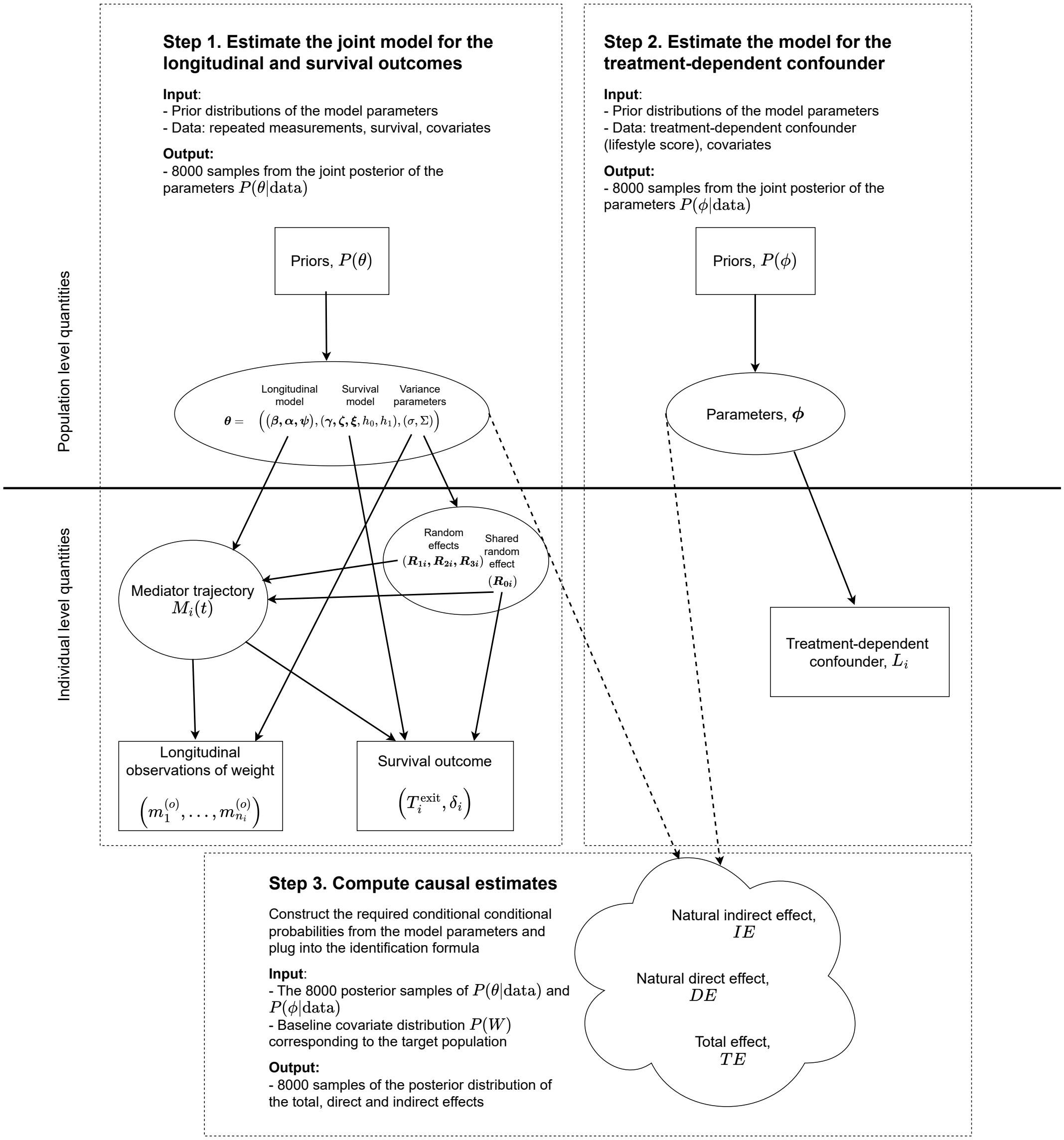

Supplementary Figure 3. Schematic illustration of the procedure used to estimate the causal effects. The rectangles represent observed and the ovals unobserved quantities. The model components above the horizontal dividing line are population level quantities, while those below pertain to an individual i. The dashed boxes in the upper-left and upper-right represent the joint model for the nodes $M(\cdot)$ and T, and the separately estimated model for the node L, respectively. For simplicity, the observed quantities (treatment and baseline covariates) are omitted from the figure.

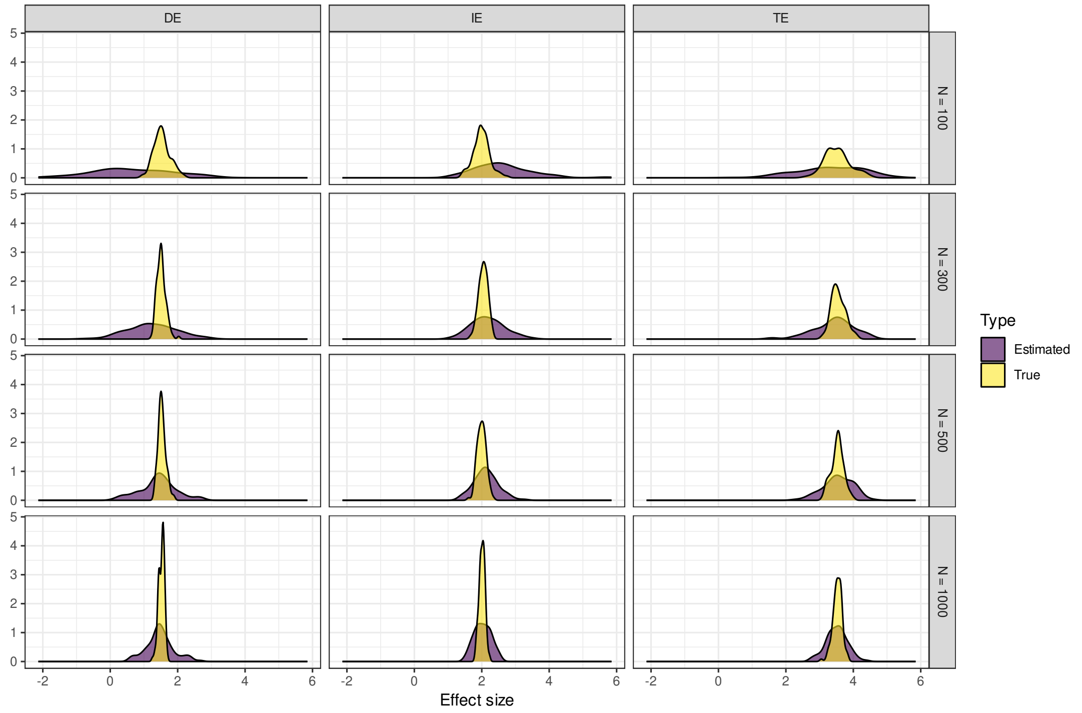

Supplementary Figure 4. Distributions of estimated causal effects (posterior means) and sample-based "true" causal effects based on 100 simulated datasets for samples sizes $N = 100, 300, 500, 1000$.

simulated according to the current change parameterisation, but for simplicity, covariates were omitted. The parameters were chosen so that both large direct and indirect effects were present.

Figure 4 shows the distributions of the estimated effects (posterior means) and the distributions of the sample-based effects, arising from sampling variability, while Figure 5 shows the distributions of the bias (difference between the estimate and the sample-based effect). With a sample sample size of 100, the distributions of the estimates are very wide and slightly biased. With a sample size of 500, the estimation can be considered satisfactory, and with 1,000 it is already very good. The sample size in the empirical application of this paper was 500, in a model that also included covariates.

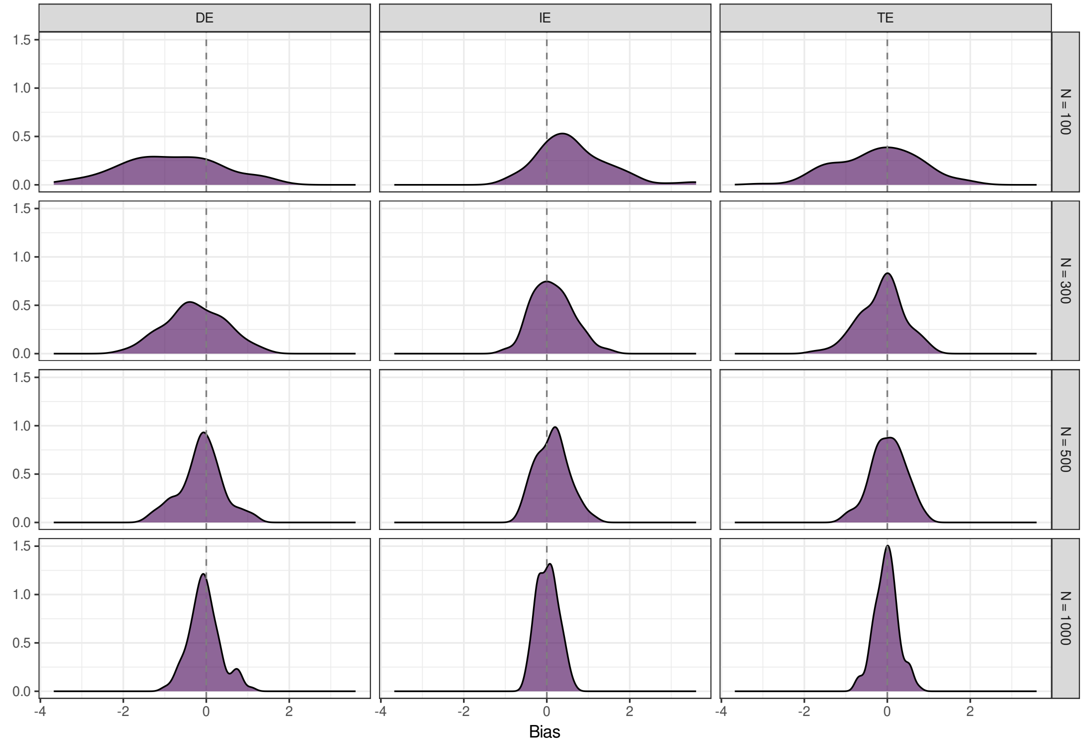

Supplementary Figure 5. Distributions of estimation bias (difference between posterior mean and sample-based effect), based on 100 simulated datasets for samples sizes $N = 100, 300, 500, 1000$.

References

1. Tchetgen Tchetgen EJ & Vanderweele TJ (2014) Identification of natural direct effects when a confounder of the mediator is directly affected by exposure. Epidemiology, 25(2):282-291.
2. Vehtari A, Gelman A, & Gabry, J (2015) Practical Bayesian model evaluation using leave-one-out cross-validation and WAIC. Statistics and Computing, 27: 1413–1432.
3. Miles C, Kanki P, Meloni S., et al. (2017) On Partial Identification of the Natural Indirect Effect. Journal of Causal Inference, 5(2): 20160004.
