# Supplementary material for: Mediation analysis in longitudinal intervention studies with an ordinal treatment-dependent confounder
Source: Stat Methods Med Res. 2026 Mar 18;35(4):773–94. doi: 10.1177/09622802261418211 (PMC13161496; doi:10.1177/09622802261418211)
Supplement: sj-zip-2-smm-10.1177_09622802261418211 - Supplemental material for Mediation analysis in longitudinal intervention studies with an ordinal treatment-dependent confounder [file sj-zip-2-smm-10.1177_09622802261418211.zip › fig_estimation.pdf]

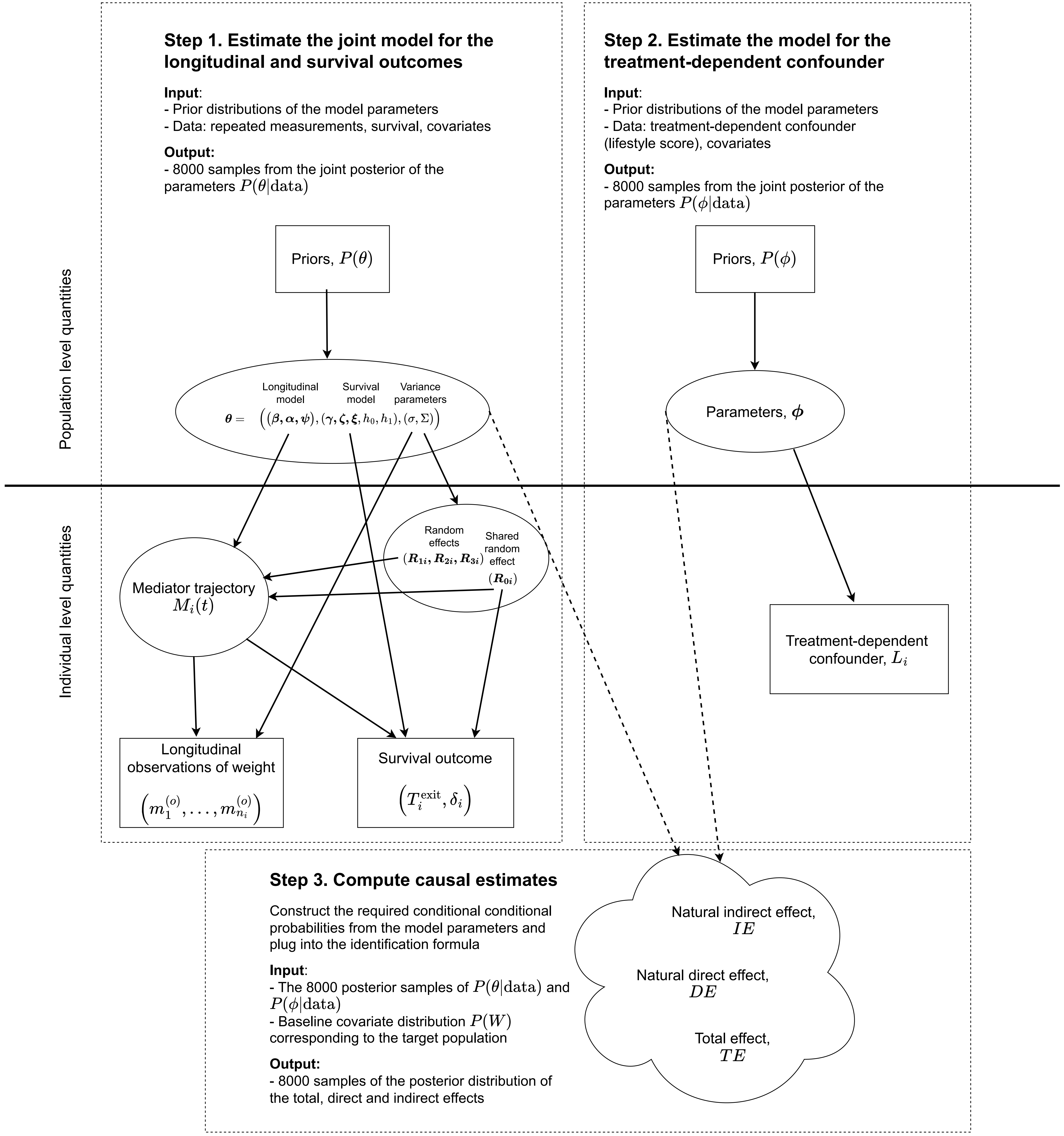

Step 1. Estimate the joint model for the longitudinal and survival outcomes
Input:
- Prior distributions of the model parameters
- Data: repeated measurements, survival, covariates
Output:
- 8000 samples from the joint posterior of the parameters $P(\theta|\mathrm{data})$
Priors, $P(\theta)$
Longitudinal model
Survival model
Variance parameters
$\boldsymbol{\theta} = \Big((\boldsymbol{\beta}, \boldsymbol{\alpha}, \boldsymbol{\psi}), (\boldsymbol{\gamma}, \boldsymbol{\zeta}, \boldsymbol{\xi}, h_0, h_1), (\sigma, \Sigma)\Big)$
Step 2. Estimate the model for the treatment-dependent confounder
Input:
- Prior distributions of the model parameters
- Data: treatment-dependent confounder (lifestyle score), covariates
Output:
- 8000 samples from the joint posterior of the parameters $P(\phi|\mathrm{data})$
Priors, $P(\phi)$
Parameters, $\boldsymbol{\phi}$
Population level quantities
Individual level quantities
Random effects
$(\boldsymbol{R_{1i}}, \boldsymbol{R_{2i}}, \boldsymbol{R_{3i}})$
Shared random effect
$(\boldsymbol{R_{0i}})$
Mediator trajectory
$M_i(t)$
Treatment-dependent confounder, L_i
Longitudinal observations of weight
$\Big(m_1^{(o)}, \ldots, m_{n_i}^{(o)}\Big)$
Survival outcome
$\Big(T_i^{\mathrm{exit}}, \delta_i\Big)$
Step 3. Compute causal estimates
Construct the required conditional conditional probabilities from the model parameters and plug into the identification formula
Input:
- The 8000 posterior samples of $P(\theta|\mathrm{data})$ and $P(\phi|\mathrm{data})$
- Baseline covariate distribution $P(W)$ corresponding to the target population
Output:
- 8000 samples of the posterior distribution of the total, direct and indirect effects
Natural indirect effect, IE
Natural direct effect, DE
Total effect, TE
