# Supplementary material for: Mediation analysis in longitudinal intervention studies with an ordinal treatment-dependent confounder
Source: Stat Methods Med Res. 2026 Mar 18;35(4):773–94. doi: 10.1177/09622802261418211 (PMC13161496; doi:10.1177/09622802261418211)
Supplement: sj-zip-2-smm-10.1177_09622802261418211 - Supplemental material for Mediation analysis in longitudinal intervention studies with an ordinal treatment-dependent confounder [file sj-zip-2-smm-10.1177_09622802261418211.zip › fig_mono_sens.pdf]

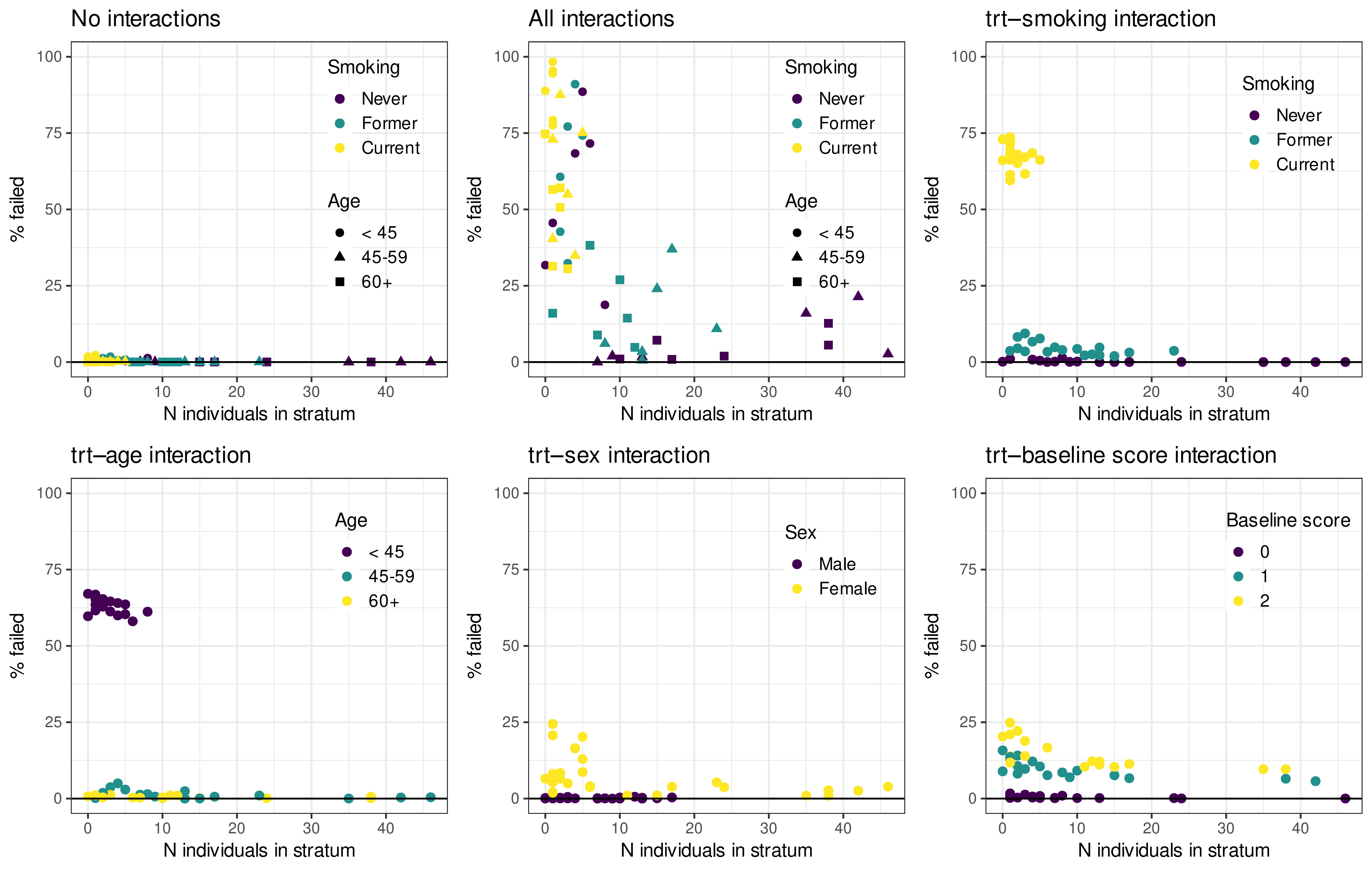

No interactions
All interactions
trt–smoking interaction
trt–age interaction
trt–sex interaction
trt–baseline score interaction
% failed
N individuals in stratum
Smoking
Never
Former
Current
Age
< 45
45-59
60+
Sex
Male
Female
Baseline score
0
1
2
0
25
50
75
100
0
10
20
30
40
