# Supplementary figures and images for: Mediation analysis in longitudinal intervention studies with an ordinal treatment-dependent confounder
Source: Stat Methods Med Res. 2026 Mar 18;35(4):773–94. doi: 10.1177/09622802261418211 (PMC13161496; doi:10.1177/09622802261418211)

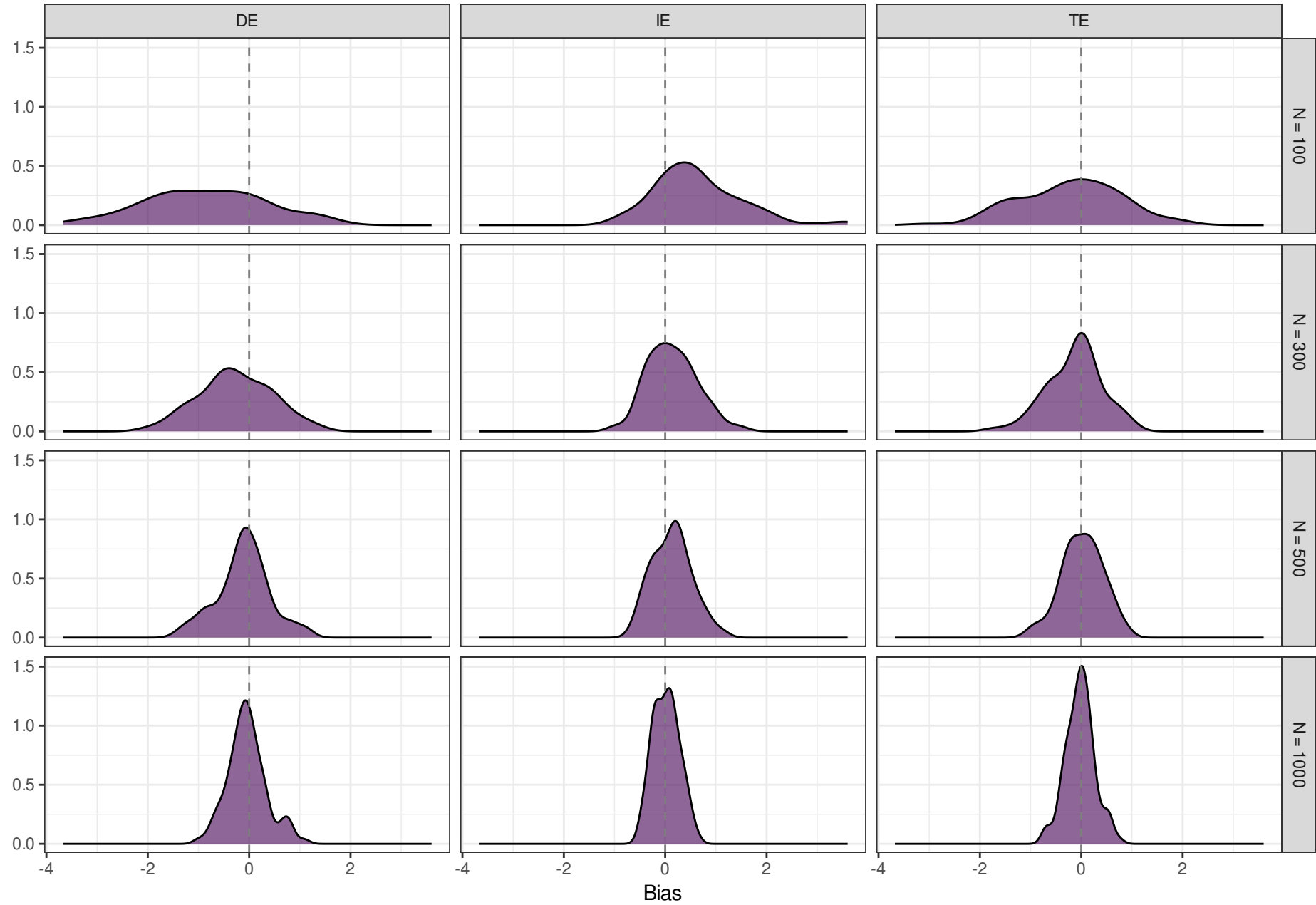

Supplement: sj-zip-2-smm-10.1177_09622802261418211 - Supplemental material for Mediation analysis in longitudinal intervention studies with an ordinal treatment-dependent confounder [file sj-zip-2-smm-10.1177_09622802261418211.zip › fig_sim2.pdf]

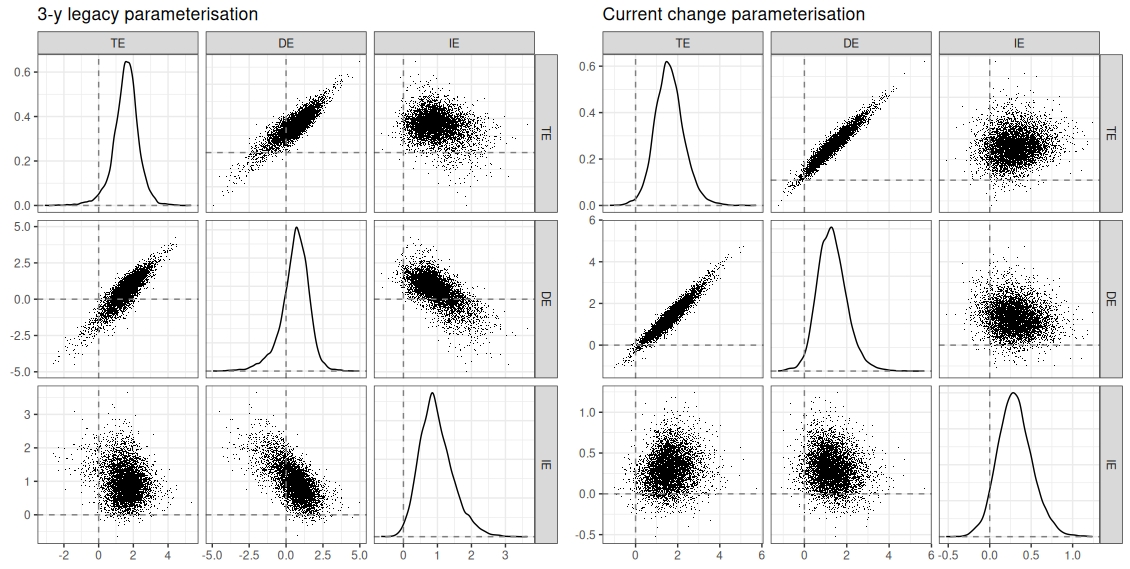

Supplement: sj-zip-2-smm-10.1177_09622802261418211 - Supplemental material for Mediation analysis in longitudinal intervention studies with an ordinal treatment-dependent confounder [file sj-zip-2-smm-10.1177_09622802261418211.zip › fig_joint_post.jpeg]

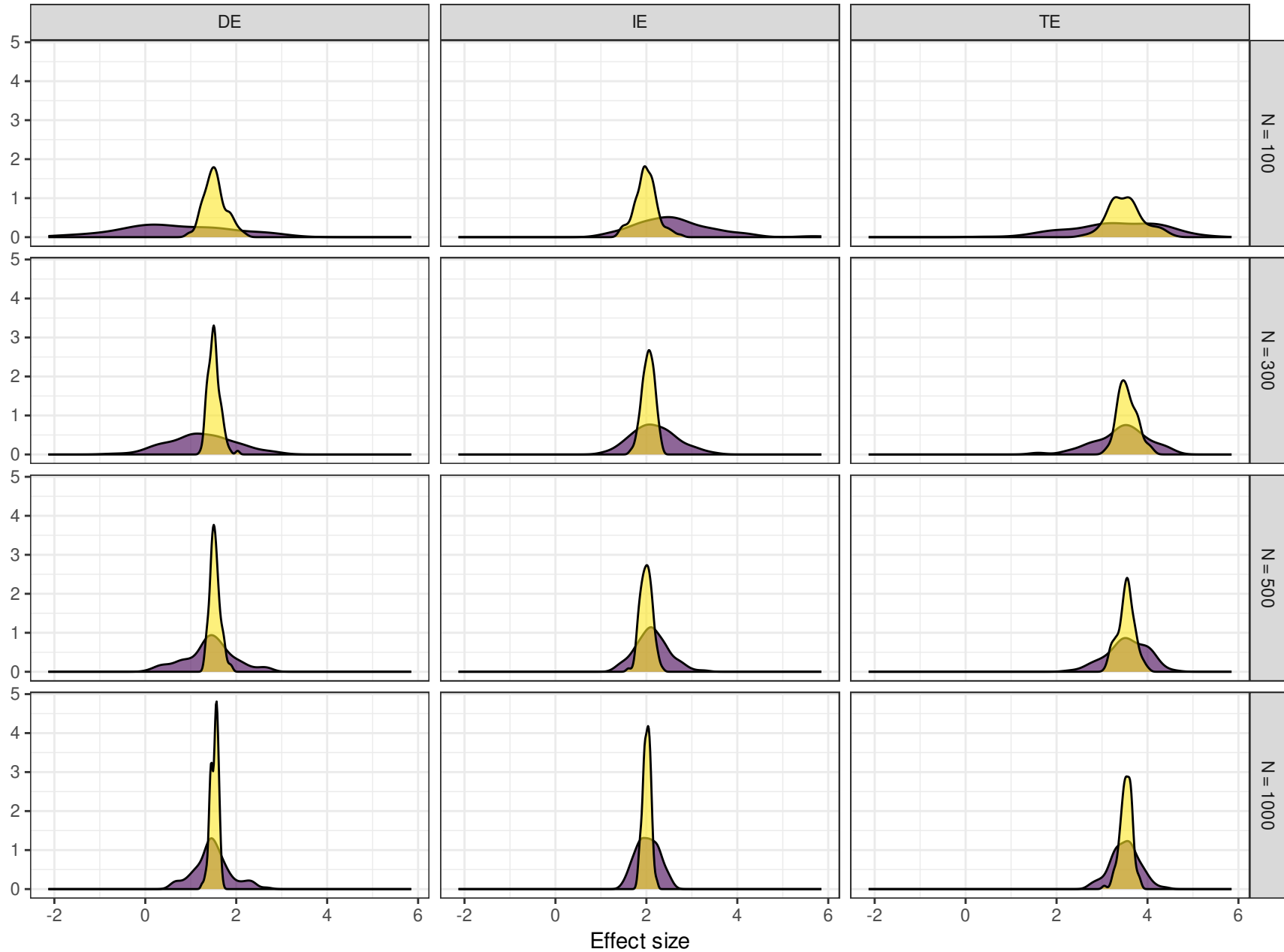

Supplement: sj-zip-2-smm-10.1177_09622802261418211 - Supplemental material for Mediation analysis in longitudinal intervention studies with an ordinal treatment-dependent confounder [file sj-zip-2-smm-10.1177_09622802261418211.zip › fig_sim1.pdf]
